# Herpes Simplex Virus 1 Entry Glycoproteins Form Complexes before and during Membrane Fusion

**DOI:** 10.1128/mbio.02039-22

**Published:** 2022-08-16

**Authors:** Zemplen Pataki, Andrea Rebolledo Viveros, Ekaterina E. Heldwein

**Affiliations:** a Department of Molecular Biology and Microbiology, Tufts University School of Medicine, Boston, Massachusetts, USA; b Graduate Program in Molecular Microbiology, Graduate School of Biomedical Sciences, Tufts University School of Medicine, Boston, Massachusetts, USA; University of North Carolina, Chapel Hill

**Keywords:** herpesvirus, HSV-1, interaction, domain, membrane fusion, glycoprotein, chimeric protein, NanoBiT protein interaction assay, split-luciferase cell-cell fusion assay

## Abstract

Herpesviruses—ubiquitous pathogens that cause persistent infections—have some of the most complex cell entry mechanisms. Entry of the prototypical herpes simplex virus 1 (HSV-1) requires coordinated efforts of 4 glycoproteins, gB, gD, gH, and gL. The current model posits that the glycoproteins do not interact before receptor engagement and that binding of gD to its receptor causes a “cascade” of sequential pairwise interactions, first activating the gH/gL complex and subsequently activating gB, the viral fusogen. But how these glycoproteins interact remains unresolved. Here, using a quantitative split-luciferase approach, we show that pairwise HSV-1 glycoprotein complexes form before fusion, interact at a steady level throughout fusion, and do not depend on the presence of the cellular receptor. Based on our findings, we propose a revised “conformational cascade” model of HSV-1 entry. We hypothesize that all 4 glycoproteins assemble into a complex before fusion, with gH/gL positioned between gD and gB. Once gD binds to a cognate receptor, the proximity of the glycoproteins within this complex allows for efficient transmission of the activating signal from the receptor-activated gD to gH/gL to gB through sequential conformational changes, ultimately triggering the fusogenic refolding of gB. Our results also highlight previously unappreciated contributions of the transmembrane and cytoplasmic domains to glycoprotein interactions and fusion. Similar principles could be at play in other multicomponent viral entry systems, and the split-luciferase approach used here is a powerful tool for investigating protein-protein interactions in these and a variety of other systems.

## INTRODUCTION

Enveloped viruses enter cells by fusing their membrane envelope with a cellular membrane, and most use a single viral protein, termed a fusogen, to perform this function. Fusogens bridge apposing membranes and merge them by refolding from the high-energy prefusion conformation into a lower-energy postfusion conformation. The energy released during these favorable conformational rearrangements is thought to overcome the activation energy of the membrane fusion process (reviewed in reference 1). Fusogens are commonly activated, or triggered, by either exposure to low pH or by binding to a receptor on the target cell (reviewed in reference 1). In herpesviruses, the fusion mechanism is more complex, however, and requires three or more viral proteins that join forces to bring about fusion (reviewed in reference 2). These double-stranded DNA viruses are significant pathogens that establish lifelong infections (reviewed in reference 3). Herpes simplex virus 1 (HSV-1), the focus of this study, is a prototypical herpesvirus (reviewed in reference 4) that infects ~67% of people under the age of 50 worldwide (5) and causes ailments ranging from oral sores (reviewed in reference 6) to encephalitis (reviewed in reference 7–9). As there is no curative treatment (reviewed in references 10) or vaccines for HSV-1 (11), a better understanding of HSV-1 biology is essential for combating the global burden of HSV-1 disease.

Glycoprotein B (gB) is the conserved fusogen in herpesviruses. By analogy with other fusogens, this homotrimeric protein is thought to merge membranes by refolding from the prefusion (12, 13) to the postfusion conformation (14–18). However, gB is not a stand-alone fusogen and must be activated by a complex of two conserved viral glycoproteins, gH and gL (although some mutant gB forms can mediate fusion independently, albeit at a reduced level [19, 20]). In some cases, such as in Kaposi Sarcoma-associated herpesvirus (KSHV) (21, 22) or varicella zoster virus (VZV) (23) or during Epstein-Barr virus (EBV) infection of epithelial cells (24), gH/gL activates gB upon binding to a cognate host cell receptor directly (reviewed in references 2 and 25). In other cases, such as HSV or human cytomegalovirus (HCMV) or during EBV infection of B cells, gH/gL instead detects the host cell receptor indirectly by binding to an accessory viral protein (reviewed in references 2 and 25), e.g., gD in HSV-1 and 2 (26, 27; reviewed in reference 28), UL128/UL130/UL131A or gO in HCMV (29–31), and gp42 in EBV (32). How these multiple glycoproteins interact to bring about fusion is a key question that has not yet been fully answered. While structures of gH/gL bound to accessory viral proteins have been determined for EBV (33) and HCMV (34, 35), and the gH/gL-gB complex in HCMV has been captured by coimmunoprecipitation (36) and visualized at low resolution (37), very little is known about how HSV-1 gD, gH/gL, and gB interact.

HSV-1 gD, gH, and gB are transmembrane proteins that consist of extracellular domains, or ectodomains, single-spanning transmembrane domains (TMD), and cytoplasmic domains (CTD or CT), whereas gL is a soluble protein that binds the gH ectodomain. Any of these domains could, in principle, mediate mutual interactions. Indeed, purified recombinant HSV-1 gD and gH/gL ectodomains have been reported to bind *in vitro* in a surface plasmon experiment (38, 39). Pairwise gD-gH/gL, gD-gB, and gH/gL-gB interactions have also been detected in live cells, by C-terminally tagging each interacting partner with a split-fluorescent protein (40, 41). However, the split-fluorescent protein approach was not used quantitatively in these studies and has yielded some contradictory results, with some reports suggesting that gH/gL-gB interaction requires the presence of gD and receptor (40, 41) and others maintaining that it does not (42). Moreover, another study using the split-fluorescent protein approach detected interactions of the HSV glycoproteins gD, gH/gL, and gB with glycoproteins from an unrelated paramyxovirus (43). This suggested that the high-affinity, irreversible interactions of the split-fluorescent protein fragments can drive nonspecific interactions between tagged proteins (43 and reviewed in reference 44). Therefore, the extent and timing of interactions between HSV-1 gD, gH/gL, and gB in live cells remains unclear.

**FIG 1:**
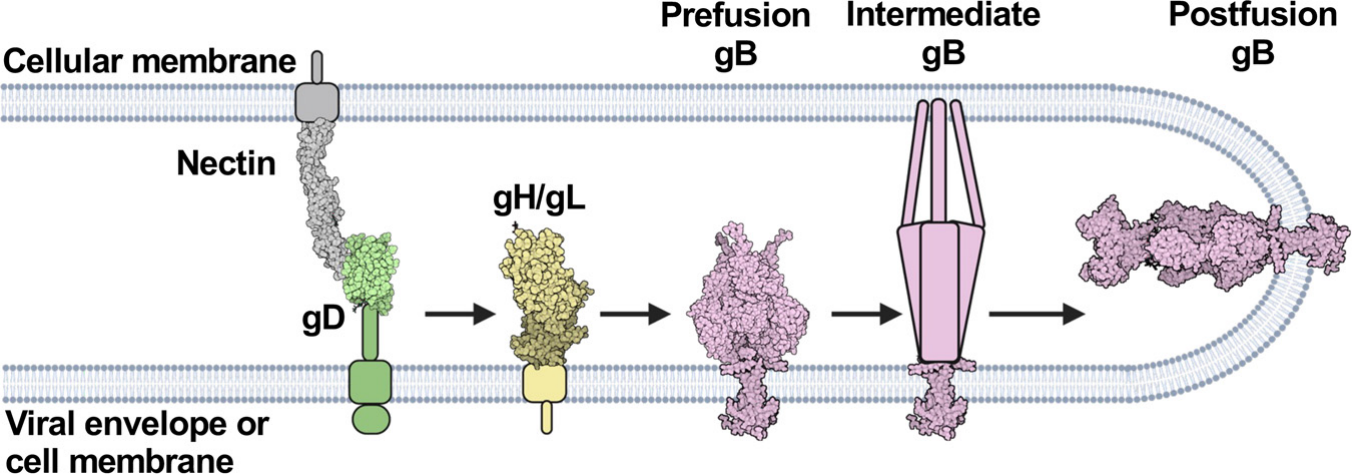
HSV-1 fusion pathway model. gD (PDB 2C36) (72) binds a receptor (PDB 3U83) (77) on the target cell. gD then activates gH/gL; gH/gL (PDB 3M1C) (78) activates gB (PDB 6Z9M and 5V2S) (12, 15) to refold and cause membrane fusion. gD is thought to be a dimer (72) but is shown here as a monomer for clarity. Figure created with BioRender.com.

Based on these prior studies, two models have emerged to explain how the glycoproteins activate one another for membrane fusion. One model is that the viral glycoproteins do not interact until gD binds to a receptor, and then gD binds to gH/gL followed by gH/gL binding to gB for sequential activation (Fig. 1) (40, 45, 46). Alternatively, gD, gH/gL, and gB are already bound to one another, and when a receptor binds to gD, activating signals are transmitted from gD to gH/gL to gB (42). In both models, the sequential activation of gD, gH/gL, and gB likely involves conformational changes of the glycoproteins (12, 15, 38, 47 and reviewed in reference 48).

Here, to clarify the timing, dynamics, and sites of interactions between gD, gH/gL, and gB, we turned to a quantitative protein-protein interaction technique called NanoBiT that uses split-luciferase fragments fused to putative interaction partners in live cells (49). Unlike split-fluorescent protein fragments, the split-luciferase fragments interact in a low-affinity, reversible manner, which reduces the likelihood of false positives and allows the detection of not only complex association but also dissociation over time.

We found that gD-gH/gL, gH/gL-gB, and gD-gB interactions were independent of the presence of the receptor nectin-1 and were steady, which suggested that these glycoprotein complexes formed before fusion and were maintained throughout fusion. By replacing the HSV-1 gH/gL domains with those of EBV gH or a scrambled sequence, we found that gD-gH/gL and gH/gL-gB interactions involved all three major domains of gH (ectodomain, TMD, and CT). However, while the HSV-1 gH TMD or CT each mediated efficient formation of the gD-gH/gL and gH/gL-gB complexes, the HSV-1 gH ectodomain did not. Therefore, the HSV-1 gH TMD and CT are more important for interactions with gD and gB than the ectodomain. In contrast, all HSV-1 gH/gL domains were essential for fusion, suggesting that glycoprotein complex formation is not sufficient for fusion. Finally, our data indicate that whereas gH and gB interact in the endoplasmic reticulum (ER), gH and gD do not.

Putting these findings together, we propose a revised model of HSV-1-mediated fusion whereby a proportion of gH/gL and gB associate in the ER and are transported to the plasma membrane together, whereas gD traffics there independently. Once at the plasma membrane, gD, gH/gL, and gB form a complex. When gD binds to a cognate receptor, the proximity of the glycoproteins within this complex allows for efficient transmission of the activating signal from gD to gH/gL and from gH/gL to gB through conformational changes, in a “conformational cascade.” Our findings increase our understanding of the HSV-1 fusion pathway and may pinpoint new targets for inhibition of HSV-1 infection.

## RESULTS

### Tagging HSV-1 gH/gL and gB with split luciferases to probe their interaction.

**FIG 2:**
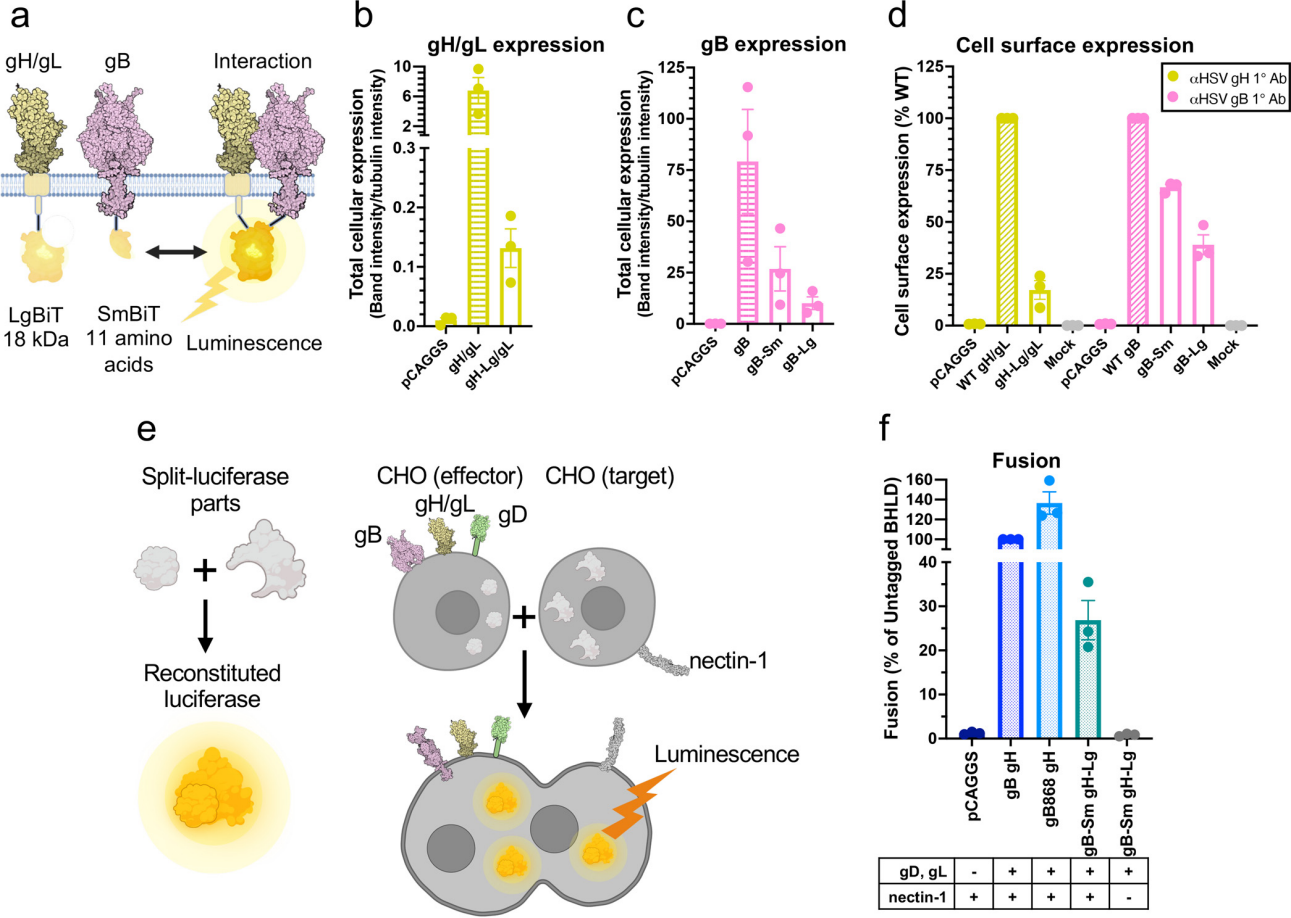
Tagging HSV-1 gH/gL and gB with split luciferases to probe their interaction. (a) NanoBiT protein-protein interaction assay approach (49). The interaction between gH/gL and gB was tested by tagging gH and gB with complementary parts of a split luciferase and transfecting into cells. Reconstitution of luciferase reports on fusion. (b to d) Total cellular expression and cell-surface expression of Lg- and Sm- tagged gH and gB by Western blotting and flow cytometry, respectively. R137 and R68 antibodies for gH/gL and gB for Western blotting, respectively. LP11 and R68 antibodies for gH/gL and gB for flow cytometry, respectively. pCAGGS was an empty vector negative control. The mock control was an untransfected negative control, incubated with a nontargeting primary antibody. Columns show the mean. Error bars are the standard error of the mean (SEM). e) Split-luciferase cell-cell fusion assay (51) experimental setup to test whether tagged proteins retain their function. Cells transfected with viral proteins fuse to cells transfected with nectin-1 receptor. Reconstitution of Rluc8 luciferase reports on fusion. (b) Total fusion of gB-Sm and gH-Lg/gL, 8 h after mixing effector and target cells. gB868 was a hyperfusogenic positive-control gB construct. pCAGGS and the condition without nectin-1 receptor are negative controls. Columns show the mean. Error bars are the SEM. Data are three biological replicates from independent experiments. Diagrams and cartoons were created using BioRender.com.

To examine the timing and duration of glycoprotein interactions in HSV-1, we turned to a split-luciferase (NanoBiT) interaction assay in live cells (49). In this assay, two proteins of interest are tagged with complementary parts of a split luciferase, Lg-BiT and Sm-BiT, and if they interact, the active luciferase is formed, and the resulting luminescence reports on the interaction (Fig. 2a). To begin with, gH and gB were C-terminally tagged with Lg-BiT or Sm-BiT (gH-Lg and gB-Sm or gH-Sm and gB-Lg). In initial studies, the gH-Lg/gB-Sm combination resulted in a higher signal-to-noise ratio in the NanoBiT assay than the gH-Sm/gB-Lg combination, so the former combination was chosen for our experiments, as recommended in the NanoBiT technical manual (50). We also used the gB-Sm/gB-Lg combination in our experiments as a positive control because gB forms trimers. gH-Lg/gL, gB-Sm, and gB-Lg had a significantly lower total cellular expression than untagged gH/gL and gB (Fig. 2b and c), with the Lg tag reducing expression more than the Sm tag. gH-Lg/gL, gB-Sm, and gB-Lg were expressed on the cell surface, however, indicating that they were properly folded and transported to the cell surface (Fig. 2d). The difference in total cellular expression between wild-type (WT) and BiT-tagged constructs was much greater than the difference in their cell surface expression. In other words, the BiT-tagged constructs had relatively high cell surface expression compared to their total cellular expression (Fig. 2b to d). In contrast, the WT gH/gL and gB constructs have a relatively low cell surface expression compared to their total cellular expression, which we attribute to protein overexpression. We note that low overall expression levels of gH-Lg/gL and gB-Sm are advantageous because they reduce the potential for nonspecific association between interaction partners (50).

To evaluate the gH/gL-gB interaction not only before but also during fusion, we tested whether gH-Lg/gL and gB-Sm could mediate membrane fusion under conditions that closely mimic the NanoBiT interaction assay conditions. We measured cell-cell fusion using a split Renilla luciferase (RLuc8) cell-cell fusion assay (51). In this assay, “effector” cells are transfected with gD, gH, gL, gB, and one part of a split luciferase, whereas “target” cells are transfected with the nectin-1 receptor and the complementary part of the split luciferase. Effector and target cells are then mixed, and cell-cell fusion is measured by the luminescence produced upon reconstitution of the luciferase (Fig. 2e). The RLuc8 luciferase used for the cell-cell fusion assay is different from the NanoBiT luciferase and uses EnduRen as a substrate instead of Endurazine. Fusion of HSV-1 gH-Lg and gB-Sm (in the presence of HSV-1 gD, gL, and nectin-1) was 27% of that of untagged HSV-1 proteins, indicating that the NanoBiT tags do not abrogate the fusion function of gH and gB (Fig. 2f). Decreased fusion extent was likely due to the lower cell surface expression of HSV-1 gH-Lg/gL (17% of the untagged HSV-1 gH/gL) and gB-Sm (67% of the untagged HSV-1 gB) (Fig. 2d). Fusion levels were similar to those of the negative control in the presence of receptor-negative target cells, which confirmed that the NanoBiT luciferase activity was not responsible for the luminescence signal measured during the cell-cell fusion assay (Fig. 2e).

### HSV-1 gH/gL and gB interact at a steady level and independently of nectin-1.

**FIG 3:**
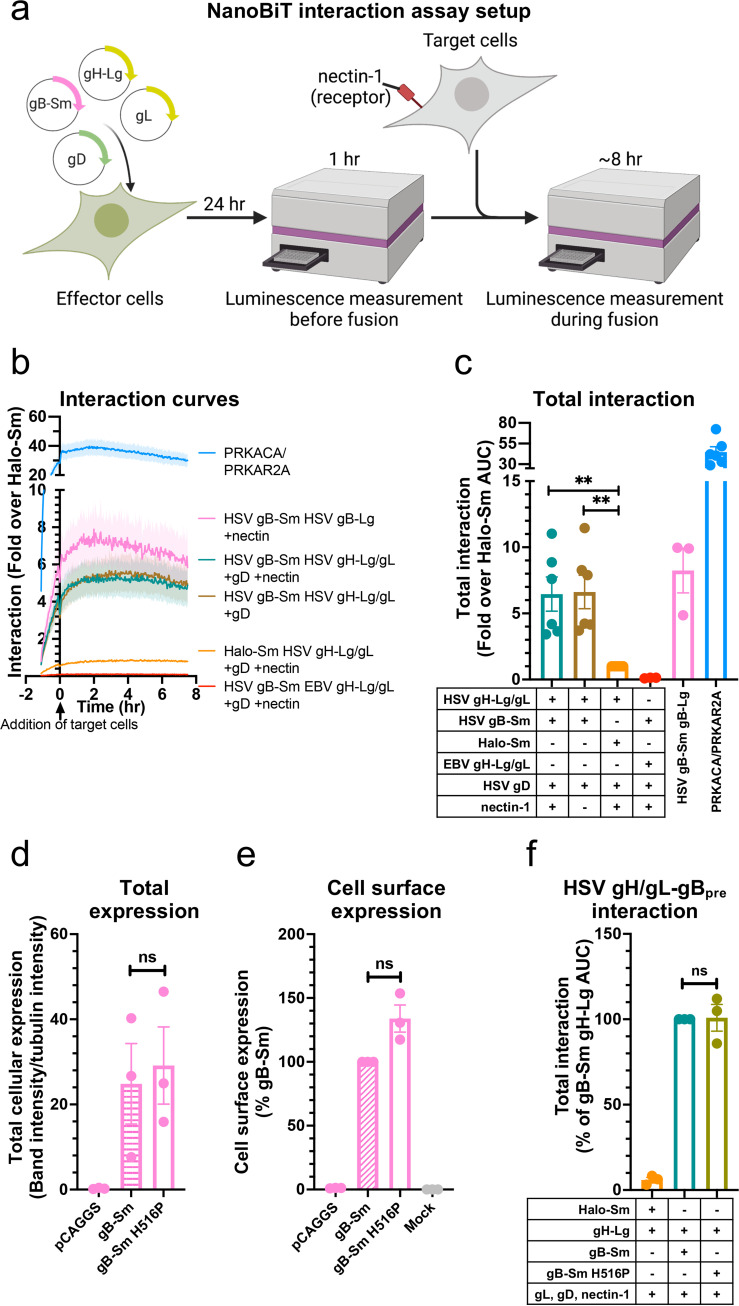
HSV-1 gH/gL and gB interact at a steady level and independently of nectin-1. (a) Interaction assay experimental setup. Cells are transfected with viral proteins required for fusion, including split-luciferase-tagged proteins of interest. Interaction is measured by luminescence before and during fusion. Fusion is induced by the addition of target cells expressing the viral receptor nectin-1. (b) gH/gL and gB interaction over time, with target cells expressing or lacking nectin-1. The Halo-Sm and EBV gH-Lg conditions were negative controls. gB-Sm/gB-Lg and PRKACA/PRKAR2A were positive controls. The shaded regions are the SEM. (c) The interactions are quantified by calculating the area under the curve (AUC). (d and e) Total cellular expression and cell-surface expression of Sm-tagged gB H516P—which locks gB in its prefusion conformation—by Western blotting and flow cytometry, respectively. (f) The interaction between gH/gL and gB H516P. Columns show the mean. Error bars are the SEM. ns, not statistically significant; **, *P* < 0.01. Data in all panels are three biological replicates from independent experiments. Diagrams were created with BioRender.com.

Interaction between gH/gL and gB was measured before and during fusion that was initiated by the addition of target cells expressing the HSV-1 receptor nectin-1 (Fig. 3a). The assay conditions were the same as for the cell-cell fusion assay except that the split RLuc8 luciferase fragments were not transfected and the NanoBiT luciferase substrate was used. Sm-BiT fused to HaloTag (Halo-Sm), which is unlikely to interact with gH, was used as a negative control in the interaction assay. HaloTag is a cytoplasmic protein derived from a bacterial haloalkane dehalogenase enzyme unrelated to viral glycoproteins (reviewed in reference 52). gH/gL from Epstein-Barr virus (EBV) was tagged with Lg-BiT as another negative control. EBV is a gammaherpesvirus that is distantly related to HSV-1, an alphaherpesvirus, so EBV gH/gL was not expected to interact with HSV-1 gB. Indeed, HSV-1 gB does not mediate cell-cell fusion when paired with EBV gH/gL and vice versa (53). HSV gB-Sm and gB-Lg were used as positive controls. NanoBiT-tagged protein kinase A catalytic (PRKACA) and type 2A regulatory (PRKAR2A) subunits, known to interact, served as other positive controls (49). Luciferase substrate was added directly before the luminescence measurements were begun. It takes a few hours after substrate addition to reach maximum luminescence signal, after which there is slight signal decay from substrate turnover (54). We found that HSV-1 gH-Lg/gL and gB-Sm interact at a steady level before and during fusion (Fig. 3b and c). gH/gL-gB interaction was detected in the absence of the target cells, and no noticeable change in gH/gL-gB interaction was seen upon addition of nectin-1-expressing target cells (Fig. 3b). Further, the extent of interaction was similar regardless of the presence of nectin-1 in target cells (Fig. 3c). We conclude that under these experimental conditions, HSV-1 gH/gL and gB form a complex that exists at a steady level and does not require gD-nectin-1 interaction. EBV gH-Lg/gL did not interact with HSV gB-Sm, whereas HSV gB-Sm interacted with gB-Lg, as expected (Fig. 3b and c).

### HSV-1 gH/gL interacts with both prefusion and postfusion forms of HSV-1 gB.

Our results suggested that gH/gL and gB interact but left unclear whether gH/gL interacted with the prefusion, postfusion, or both conformations of gB because gB exists as a mixture of these two conformations on the cell surface (55–57) and on virions (58). Therefore, we introduced the H516P mutation into gB-Sm, which has been reported to stabilize gB in its prefusion conformation (12). The gB-Sm H516P mutant had similar total cellular expression as the WT gB-Sm (Fig. 3d) and was expressed on the cell surface (Fig. 3e). The gB-Sm H516P mutant interacted with gH/gL to a similar extent as the WT gB-Sm (Fig. 3f), indicating that gH/gL can interact with prefusion gB, and therefore, that the gH/gL-gB interaction occurs before fusion. Although there is no known gB mutation that stabilizes its postfusion conformation, we hypothesize that gH/gL likely also interacts with postfusion gB because the gH/gL-gB interaction remains at a steady level as fusion progresses, even as the prefusion form converts into the postfusion form.

### All HSV-1 gH domains are involved in interactions with HSV-1 gB, but the TMD and the CT are more important than the ectodomain.

**FIG 4:**
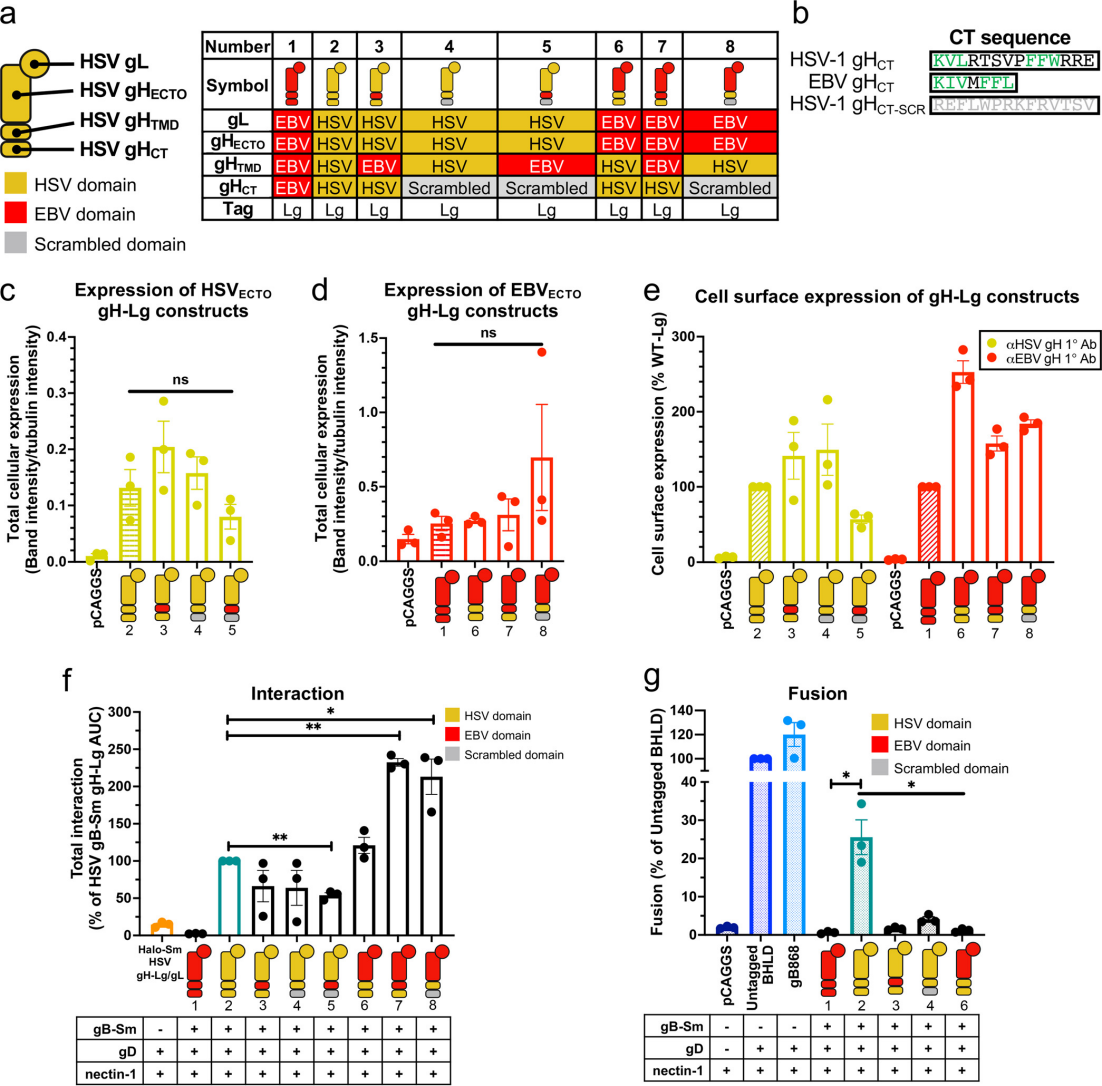
All HSV-1 gH domains are involved in interactions with HSV-1 gB. (a) Summary of the composition of gH-Lg/gL constructs designed to disrupt domain interactions. (b) Sequence comparison of HSV-1, EBV, and scrambled HSV-1 gH cytotails. Green indicates similar clusters of residues in HSV-1 and EBV. (c and d) Total cellular expression of gH-Lg/gL constructs with disrupted domain interactions compared to HSV gH-Lg/gL (2) or EBV gH-Lg/gL (1). Cartoons represent gH-Lg/gL constructs, indicating which domains are HSV-1 (yellow), EBV (red), or scrambled (gray). R137 and R2267 antibodies were used against constructs with HSV and EBV ectodomains, respectively. (e) Cell surface expression of gH-Lg/gL constructs with disrupted domain interactions. LP11 and AMMO1 antibodies were used against constructs with HSV and EBV ectodomains, respectively. (f) Interaction between gB and gH/gL constructs with disrupted ectodomain, TMD, or CTD interactions. (g) Total fusion of gB-Sm and gH-Lg/gL constructs with disrupted domain interactions 8 h after mixing effector and target cells. Columns show the mean. Error bars are the SEM. *, *P* < 0.05; **, *P* < 0.01. Data in all panels are three biological replicates from independent experiments. Cartoons were created using BioRender.com.

Having observed a steady gH/gL-gB interaction, we sought to identify the gH domains that were involved in it. Toward this goal, we created gH-Lg variants to disrupt interactions between domains. To disrupt interactions between the ectodomains or TMDs, we replaced the ectodomain or TMD of HSV-1 gH with those of gH from EBV (Fig. 4a, constructs 6 and 3, respectively). EBV is a gammaherpesvirus that is distantly related to HSV-1, an alphaherpesvirus, so EBV gH/gL is not expected to interact with HSV-1 gB. To enable proper folding, gH chimeras containing HSV-1 gH ectodomain were coexpressed with HSV-1 gL, whereas those containing EBV gH ectodomain were coexpressed with EBV gL. To disrupt interactions between the gB CTD and gH CT, the HSV-1 gH CT was scrambled by arranging its amino acids in a random order (Fig. 4a, construct 4). This method was chosen instead of replacing the HSV-1 gH CT with that of EBV gH because we reasoned that the scrambled HSV-1 gH CT was less likely to interact with the HSV-1 gB CTD than the EBV gH CT, which shares sequence similarity with the HSV-1 gH CT (Fig. 4b). For example, the EBV gH CT has two hydrophobic amino acid clusters, KIV and FFL, which are similar to the KVL and FFW clusters in the HSV-1 gH CT (Fig. 4b). Additional constructs were also generated to disrupt interactions in two domains simultaneously (Fig. 4a, constructs 5, 7, and 8). Finally, the full-length EBV gH-Lg/gL construct was again used as a negative control (Fig. 4a, construct 1).

To rule out the possibility that differences in the expression levels of the gH-Lg/gL constructs could account for differences in their interactions with HSV-1 gB, we measured their total cellular expression. Total cellular expression was measured because gH/gL and gB may interact not only on the cell surface but also at intracellular locations, e.g., in the ER and Golgi. Indeed, it has been shown that gB and gH/gL from HCMV, a betaherpesvirus, can interact in the ER (36). The total cellular expression of the gH-Lg/gL constructs was not statistically significantly different from HSV-1 gH-Lg/gL and EBV gH-Lg/gL (Fig. 4c and d), so differences in protein expression are not expected to account for any differences in interaction with HSV-1 gB among the constructs. All constructs were expressed on the cell surface (Fig. 4e), suggesting that they were properly folded.

When single HSV-1 gH domains were replaced, interactions either remained at a WT HSV-1 gH-Lg/gL level (ECTO_EBV_; Fig. 4f, construct 6) or were reduced to ~60%, albeit not to a statistically significant extent (TMD_EBV_ and CT_SCR_ constructs; Fig. 4f, constructs 3 and 4). We then tested gH constructs in which two domains were replaced simultaneously. When both the gH TMD and CT were replaced (TMD_EBV_-CT_SCR_; Fig. 4f, construct 5), interaction decreased to 54%, indicating that when only the gH ectodomain is from HSV-1, gH/gL-gB interaction cannot be maintained at the WT level. Surprisingly, when both the gH ectodomain and TMD, or both the gH ectodomain and CT, were replaced simultaneously, interactions increased ~2-fold (ECTO_EBV_-TMD_EBV_ and ECTO_EBV_-CT_SCR_; Fig. 4f, constructs 7 and 8). We conclude that although all gH domains appear to be involved in interactions with gB, when only the TMD or the CT is from HSV-1, they are sufficient to maintain WT-level interactions, whereas the ectodomain is not.

### All three gH domains are required for fusion.

While the TMD or the CT of HSV-1 gH appears sufficient for maintaining WT-level interactions with gB when the rest of the domains are replaced with their EBV gH counterparts or scrambled, it is unlikely that these HSV-1 domains would be sufficient for fusion. To examine this, we tested some of the gH-Lg constructs described above for their ability to support fusion using the cell-cell fusion assay (51). Unlike the WT HSV-1 gH-Lg/gL (Fig. 4g, construct 2), none of the gH-Lg/gL mutant constructs (Fig. 4g, constructs 3, 4, and 6) were able to support fusion. Thus, interaction between gH/gL and gB is not sufficient for fusion, and all three HSV-1 gH domains are required for fusion.

### HSV-1 gH/gL and gD interact at a steady level and independently of nectin-1.

**FIG 5:**
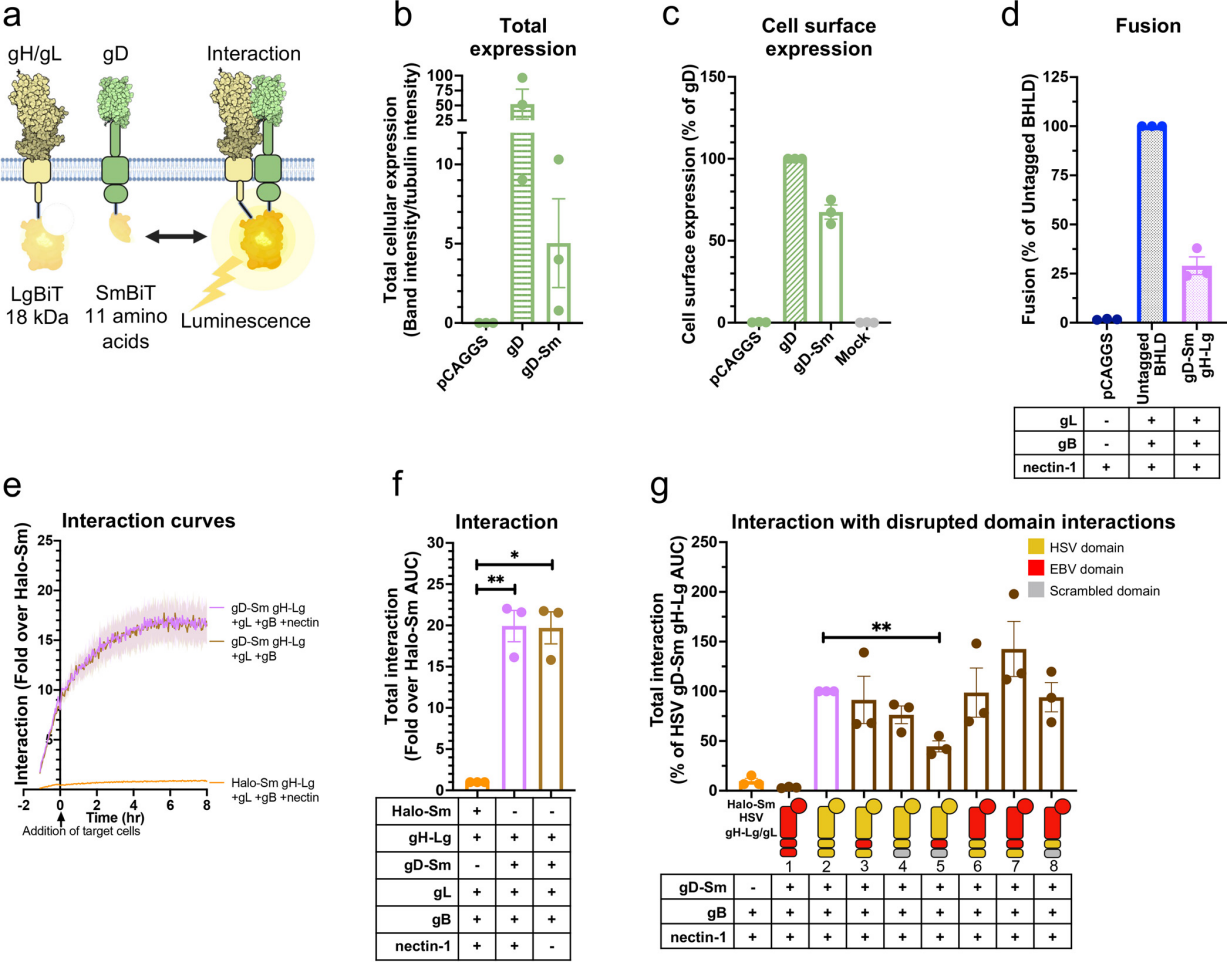
HSV-1 gH/gL and gD interact at a steady level and independently of nectin-1, through all domains. (a) NanoBiT interaction assay setup to test gD-gH/gL interactions. (b and c) Total cellular expression and cell surface expression of gD-Sm by Western blotting and flow cytometry, respectively. R7 and DL6 antibodies were used, respectively. (d) Total fusion of gD-Sm and gH-Lg/gL. (e and f) Interaction of gD and gH/gL. Curves or bars represent the mean, and the shaded area or error bars are the SEM. (g) Interaction of gD with gH/gL with disrupted domain interactions. Columns are the mean, and error bars are the SEM. *, *P* < 0.05; **, *P* < 0.01. Data in all panels are three biological replicates from independent experiments. Illustrations were created with BioRender.com.

The regulatory cascade model of HSV-1-mediated membrane fusion (Fig. 1) is predicated upon both gH/gL-gB and gD-gH/gL interactions. To probe the gD-gH/gL interaction and the role of the receptor in it, we generated the gD-Sm construct to test its interaction with gH-Lg/gL (Fig. 5a). gD-Sm had reduced total cellular expression relative to the untagged gD (Fig. 5b). This is consistent with the expression levels of other NanoBiT-tagged constructs and is advantageous because low expression reduces the likelihood of nonspecific interactions. gD-Sm was expressed on the cell surface at 68% of the untagged HSV-1 gD (Fig. 5c) and supported fusion in combination with gH-Lg at 29% of that of untagged HSV-1 proteins (Fig. 5d), indicating that the NanoBiT tag does not abrogate the fusion function of gD. We found that gD-Sm interacted with gH-Lg/gL at a steady level before and during fusion and that the presence of nectin-1 had no apparent effect on the interaction (Fig. 5e and f). We conclude that under these experimental conditions, HSV-1 gD and gH/gL form a complex that exists at a steady level and does not require gD-nectin-1 interaction.

### All HSV-1 gH domains are involved in interactions with HSV-1 gD, but the TMD and the CT are more important than the ectodomain.

To identify gH domains important for the gD-gH/gL interaction, we tested the interaction of gD-Sm with the same series of gH-Lg/gL constructs that were used to probe the gH/gL-gB interaction. As expected, EBV gH-Lg/gL did not interact with HSV-1 gD-Sm (Fig. 5g). All but one gH-Lg construct interacted with HSV-1 gD-Sm at a WT HSV-1 gH-Lg level, with no statistically significant differences. Only the construct in which both the gH TMD and CT were replaced (TMD_EBV_-CT_SCR_; Fig. 5g, construct 5) had a significantly decreased interaction, to 45% of that of WT HSV-1 gH-Lg. Thus, we conclude that although all gH domains are involved in interactions with gD, the TMD or the CT from HSV-1 are sufficient to maintain WT-level interactions, whereas the ectodomain is not.

### HSV-1 gD and gB interact at a steady level and independently of nectin-1.

**FIG 6:**
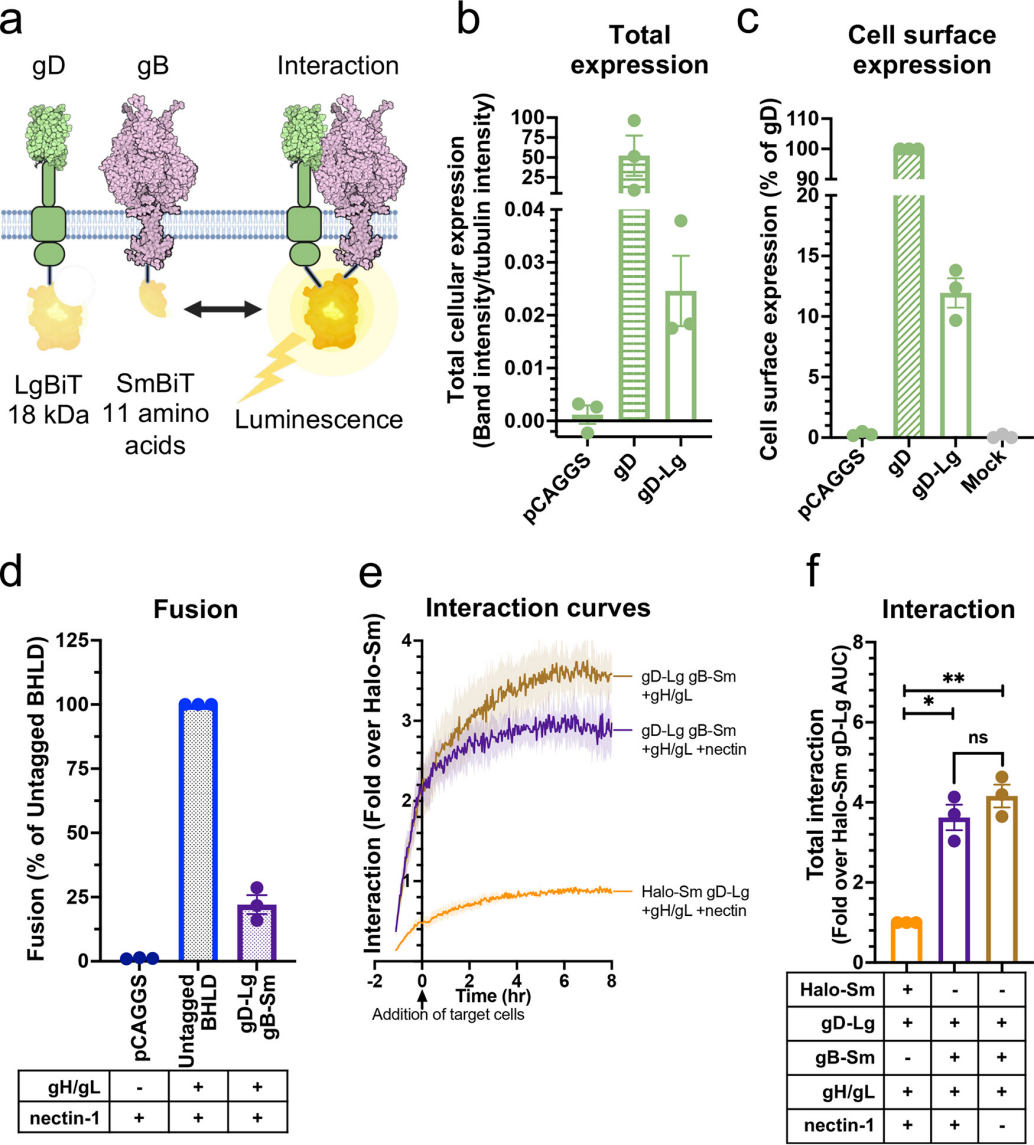
gD and gB interact at a steady level and independently of nectin-1. (a) NanoBiT interaction assay setup to test gD-gB interactions. Created with BioRender.com. (b and c) Total cellular expression and cell surface expression of gD-Lg by Western blotting and flow cytometry, respectively. (d) Total fusion of gD-Lg and gB-Sm. (e and f) Interaction of gD and gB. Curves indicate the mean and the shaded area is the SEM. Columns are the mean and error bars are the SEM. *, *P* < 0.05; **, *P* < 0.01. Data in all panels are three biological replicates from independent experiments.

Since HSV-1 gH/gL interacted at a steady level with both HSV-1 gB and gD, we asked whether gD and gB could also interact. Thus, we generated HSV-1 gD-Lg to test its interaction with gB-Sm (Fig. 6a). Similarly to the tagged gH and gB constructs, gD-Lg had low total cellular expression (Fig. 6b), was expressed on the cell surface at 12% of the untagged HSV-1 gD (Fig. 6c), and supported cell-cell fusion in combination with gB-Sm at 22% of that of untagged HSV-1 proteins (Fig. 6d). We found that gD-Lg interacted with gB-Sm at a steady level before and during fusion and that the presence of nectin-1 had no apparent effect on the interaction (Fig. 6e and f). We conclude that under these experimental conditions, HSV-1 gD and gB form a complex that exists at a steady level and does not require gD-nectin-1 interaction.

### HSV-1 gD, gH/gL, and gB compete with one another for binding.

**FIG 7:**
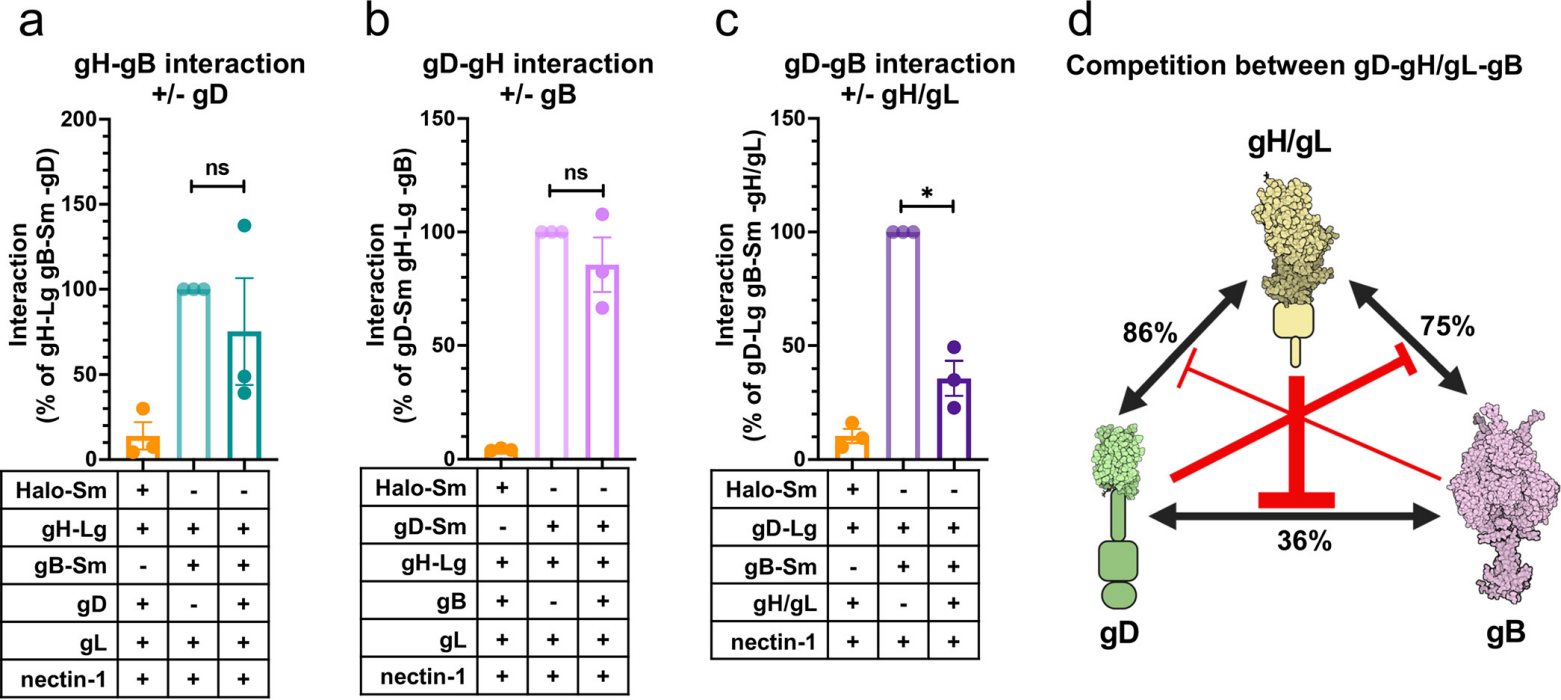
gD, gH/gL, and gB compete with one another for binding. (a) Interaction of gH/gL and gB in the absence versus the presence of gD. (b) Interaction of gD and gH/gL in the absence versus the presence of gB. (c) Interaction of gD and gB in the absence versus the presence of gH/gL. Columns indicate the mean and error bars are the SEM. *, *P* < 0.05. Data represent three biological replicates from independent experiments in all panels. (d) Binding competition model between gD, gH/gL, and gB. The interaction of each pair was decreased in the presence of the third interacting partner, suggesting interaction inhibition by binding competition. The sizes of the red inhibitory arrows are proportional to the degree of inhibition. Values indicate the percentage of the interaction of two interacting partners that remains after inhibition by the presence of the third interacting partner. Created using BioRender.com.

So far, we have shown that HSV-1 gD, gH/gL, and gB interact with one another in a pairwise manner. However, in all these experiments, all four HSV-1 glycoproteins were present, leaving unclear whether these pairwise interactions required the presence of the third partner. For example, the gD-gB interaction could require the presence of gH/gL. Alternatively, gD, gH/gL, and gB could compete with one another for binding. To differentiate these possibilities, we tested all pairwise interactions in the absence of the third interacting partner. We detected all three complexes, gD-gH/gL, gH/gL-gB, and gD-gB, even in the absence of the third interacting partner (Fig. 7a to c). Moreover, we found that all three interactions were reduced in the presence of the third partner, but to a different extent. Whereas gD-gH/gL and gH/gL-gB interactions were minimally reduced in the presence of gB or gD, respectively (Fig. 7a and b), the gD-gB interaction was reduced ~3-fold in the presence of gH/gL (Fig. 7c). The observed inhibitory effects are likely due to competition for binding (Fig. 7d). Nonetheless, we cannot exclude the possibility that changes in interaction were due to changes in total cellular expression of the glycoproteins in the presence versus the absence of the third interacting partner.

### HSV-1 gL is important for gH-gB and gD-gH interactions and for cell surface expression of gH and gB.

**FIG 8:**
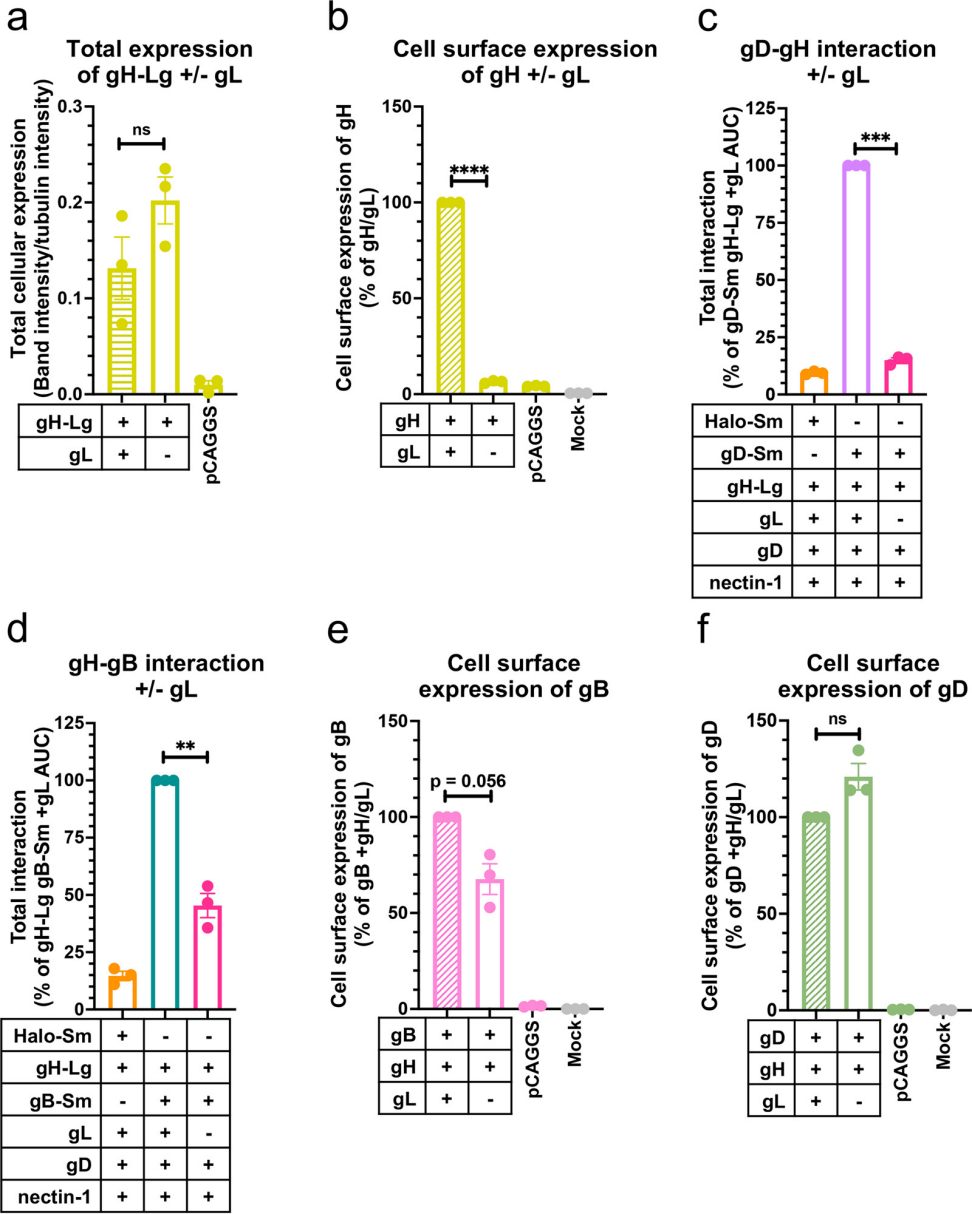
HSV-1 gL is required for gH-gB and gD-gH interactions and for cell surface expression of gH and gB. (a) Total cellular expression of gH-Lg in the presence versus the absence of gL. (b) gH cell surface expression in the presence versus the absence of gL (60, 70) using R137. (c) gD-gH interaction in the presence versus the absence of gL. (d) gH-gB interaction in the presence versus the absence of gL. (e) gB cell surface expression in the presence versus the absence of gL. (f) gD cell surface expression in the presence versus the absence of gL. Columns are the mean. Error bars are the SEM. **, *P* < 0.01; ***, *P* < 0.001; ****, *P* < 0.0001. Data represent three biological replicates from independent experiments in all panels.

gL is thought to be required for transport of gH to the cell surface (59). We confirmed that the total cellular expression levels of gH-Lg were similar in the presence or absence of gL (Fig. 8a) and that gH was not expressed on the cell surface without gL (Fig. 8b). Next, we examined the location of gD-gH and gH-gB interactions. In the absence of gL, gH localizes to the ER (60). Therefore, if gD-gH and gH-gB interactions occurred only on the cell surface, they would no longer be detectable in the absence of gL. Conversely, if interactions occurred in the ER, they could be maintained in the absence of gL. We found that, in the absence of HSV-1 gL, the gD-gH interaction reduced nearly to background levels, ~6-fold (Fig. 8c), which suggested that gD and gH/gL interact mainly on the cell surface. In contrast, in the absence of gL, the gH-gB interaction decreased ~2-fold (Fig. 8d), suggesting that gH and gB can interact in the ER at ~50% of the level of gH/gL-gB interactions under our experimental conditions.

**FIG 9:**
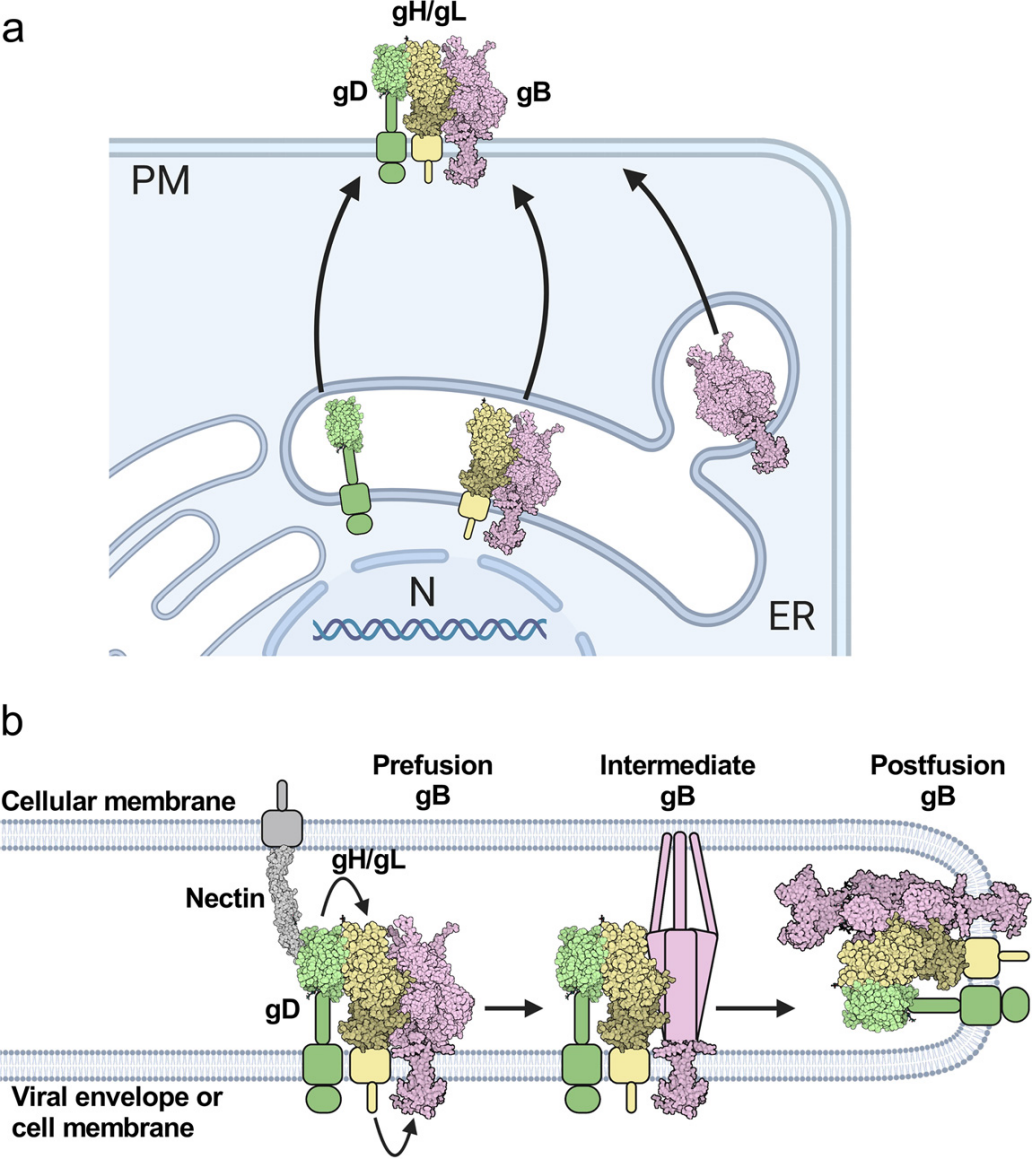
gD-gH/gL-gB trafficking, interaction, and fusion models. (a) Intracellular interactions and trafficking of gD, gH/gL, and gB. gD does not interact with gH in the ER and traffics independently of gH to the plasma membrane. gB interacts with gH in the ER and may traffic with gH/gL to the plasma membrane. Some gB traffics to the plasma membrane without gH/gL. gD, gH/gL, and gB all interact with one another once they leave the ER and compete with one another for binding. gH/gL inhibits binding of the other two binding partners the most, suggesting it binds well to both gD and gB and may position itself between gD and gB in the putative gD-gH/gL-gB complex. gH/gL interacts with gD and gB through all three domains. Cell and glycoprotein sizes are not to scale. (b) New HSV-1 fusion pathway model. gD, gH/gL, and gB are all interacting with each other before fusion. Nectin-1 binds to gD, causing a conformational change (not shown), which activates gH/gL via their ectodomains. gH/gL undergoes a conformational change (not shown) and the gH_CT_ activates the gB_CTD_. The gB ectodomain refolds and catalyzes membrane fusion. gD, gH/gL, and gB continue to interact. For this model we assume a complex with a 1:1:1 ratio of gD:gH/gL:gB, but the true stoichiometry is unknown.

Since in the absence of gL, gH is unable to leave the ER yet can still interact with gB, we next tested whether the gH-gB interaction can also prevent gB from leaving the ER to traffic to the cell surface. Indeed, the gB cell surface expression decreased to 68% in the presence of gH alone relative to when both gH and gL were present (Fig. 8e). In contrast, the gD cell surface expression was not noticeably affected by the absence of gL (Fig. 8f). We conclude that gH and gB can interact in the ER and that gH/gL and gB may traffic together to the cell surface, whereas gH and gD do not interact in the ER to any significant extent and traffic to the surface independently from one another (Fig. 9a).

## DISCUSSION

### Interactions between gD, gH/gL, and gB occur before and during fusion.

Using the NanoBiT interaction assay, which uses split luciferase (49), we established the timing, duration, and dynamics of the interactions between HSV-1 gD, gH/gL, and gB. The advantage of the NanoBiT interaction assay over the commonly used split-fluorescent proteins is that the split luciferase fragments interact in a low-affinity, reversible manner. This reduces the likelihood of false positives and allows the detection of not only complex association but also dissociation over time. The NanoBiT interaction assay has been used to study transmembrane proteins (61), but as far as we know, this study is the first application of this approach to probing interactions between viral proteins or glycoproteins. The split-luciferase approach is thus a powerful tool that may be broadly applicable across a variety of systems.

We found that pairwise gD-gH/gL, gH/gL-gB, and gD-gB interactions occurred before fusion, remained at a steady level throughout fusion, and were independent of the presence of the receptor nectin-1. As an example, gH/gL interacted with gB in the absence of gD (Fig. 7a) and also interacted with the gB H516P mutant that has been reported to lock gB in the prefusion conformation (12) (Fig. 3f). These findings support the notion that gH/gL-gB interactions occur before fusion (42). The NanoBiT signal for all three pairs, gD-gH/gL, gH/gL-gB, and gD-gB, remained at a steady level upon addition of the receptor-expressing target cells and for the next ~8 h, suggesting that the complexes do not dissociate as gB refolds from the prefusion to the postfusion conformation (Fig. 9b). Therefore, we conclude that the gD-gH/gL, gH/gL-gB, and gD-gB complexes form independently of fusion and are maintained throughout the fusion process.

The detection of gD-gH/gL, gH/gL-gB, and gD-gB interactions using the split-luciferase assay is consistent with previous studies that observed these interactions using split fluorescent proteins (40, 41). However, our observation that the gH/gL-gB interaction occurs before fusion agrees with some reports using split fluorescent proteins (42) but not others (40, 41). We hypothesize that this could be due to a higher sensitivity of the NanoBiT split-luciferase approach used here for detecting interactions (49 and reviewed in reference 44). Alternatively, gD-receptor binding could increase the rate of gH/gL-gB association/dissociation, which could increase the signal due to the irreversibility of the split-fluorescent protein interactions, explaining previous observations (40).

### gD-gH/gL and gH/gL-gB interactions involve all HSV-1 gH domains, but the TMD and CT are more important than the ectodomain, and all three domains are required for fusion.

Replacing domains of the HSV-1 gH with those of EBV gH or a scrambled sequence revealed that all three domains were involved in gD-gH/gL and gH/gL-gB interactions. For example, when the TMD or CT were the only endogenous HSV-1 gH domains, the chimeric gH could maintain WT-level interactions with HSV-1 gD and gB. However, when the ectodomain was the only endogenous HSV-1 gH domain, interaction with HSV-1 gD and gB decreased ~2-fold. Therefore, gD-gH/gL and gH/gL-gB interactions through their TMDs and CTDs are greater than those through their ectodomains. Whereas previous studies focused mainly on ectodomain interactions (38, 39, 62), our study highlights the previously overlooked yet important contributions of the TMD and CTDs to HSV-1 glycoprotein interactions. Indeed, our recent study proposed a mechanism by which the cytoplasmic tail of gH activates gB through its CTD (63).

Surprisingly, when the TMD or CT were the only endogenous HSV-1 gH domains present, the gH/gL-gB interaction was enhanced ~2-fold (Fig. 4f). One possible explanation for this could be a competition for binding of gB to the HSV-1 gH/gL ectodomain by an unknown host protein. Such a competitor would be unable to bind the constructs containing the EBV gH/gL ectodomain, which could explain increased binding of those constructs to HSV-1 gB relative to WT HSV-1 gH/gL. Indeed, integrins have been reported to bind HSV-1 gH/gL (64, 65).

While each of the HSV-1 gH/gL domains, to a certain extent, could maintain interactions with gD and gB, replacing any of the HSV-1 gH/gL domains with those of EBV gH or a scrambled sequence significantly reduced fusion (Fig. 4g). These findings suggest that all three HSV-1 gH/gL domains are required for fusion and that they each perform a specific function in fusion beyond interaction with gD and gB, such as transduction of the triggering signal from gD to gB.

### gH/gL blocks gD-gB interactions.

We found that the gD-gH/gL, gH/gL-gB, and gD-gB interactions could occur in the absence of the third interacting partner. Moreover, these pairwise interactions were greater when the third partner was absent (Fig. 7a to c), which suggested that each interacting partner inhibited the interactions of the other two partners. Since all three glycoproteins interact with one another, this inhibitory effect could be due to competition based on shared or overlapping binding sites, allosteric effects, or steric crowding. Interestingly, the inhibitory effect of gD on the gH/gL-gB interaction and gB on the gD-gH/gL interaction was relatively modest and statistically insignificant. In contrast, gH/gL caused a statistically significant, ~3-fold reduction in gD-gB interaction. This suggests that gD and gB preferentially bind to gH/gL rather than to each other, which positions gH/gL in between gD and gB in a putative gD-gH/gL-gB complex and supports the role of gH/gL as the middleman between gD and gB (reviewed in reference 66).

### gD and gB differ in their trafficking and interactions with gH/gL.

HSV-1 gD, gH/gL, and gB are produced in the ER, traffic to the plasma membrane, and become endocytosed, ending up in vesicles derived from the endosomes or *trans*-Golgi network that serve as virion assembly sites during infection (67, 68). Alphaherpesvirus glycoprotein traffic from the ER to the plasma membrane is thought to occur by the exocytic pathway (69), but whether HSV-1 gD, gH/gL, and gB interact during this process is unknown. Once at the plasma membrane, HSV-1 gB can endocytose independently of other viral glycoproteins to reach the virion assembly site, whereas gH/gL and gD require colocalization—and likely interaction—with other glycoproteins such as gM because they lack an endocytic signal (68).

Given the extensive interactions between gD, gH/gL, and gB that we observed in this study, we asked where in the cell they interact and whether these proteins cotraffic to the cell surface. By taking advantage of the inability of gH to leave the ER without gL, we found that in the absence of gL, gD-gH interactions in the ER were minimal, whereas gH-gB interactions in the ER were about 50% of the level of overall gH/gL-gB interactions (Fig. 8c and d). This suggests that gB interacts with gH to a far greater extent in the ER than gD does. In addition, we found that when gH was expressed without gL, gD cell surface expression was unaffected, while gB cell surface expression was reduced. This further supports the hypothesis that gD does not interact with gH in the ER to an appreciable degree because gD is able to leave the ER and traffic to the cell surface unimpeded. In contrast, roughly a third of gB molecules appear to be held back from trafficking to the cell surface due to interactions with gH in the ER. Therefore, we propose that a portion of gH/gL and gB interact in the ER and traffic together to the cell surface in addition to some gB that traffics independently, whereas gD traffics to the cell surface independently and then joins the gH/gL-gB complex on the cell surface (Fig. 9a).

Both gD and gB interact with gH/gL through their ectodomains, TMDs, and CTDs, so it is interesting that gB but not gD interacted with gH in the ER. How is gD able to bind to gH/gL on the cell surface but avoid binding to gH in the ER? It is possible that the gD-gH interaction is more dynamic than the gH-gB interaction, with more frequent dissociation, allowing gD to gradually escape the ER without gH/gL. Another possibility is that the conformation of gH is different in the absence of gL than when it is complexed with gL, which has been suggested in several studies (59, 70, 71), causing decreased gD-gH interaction relative to gD-gH/gL interactions.

### New HSV-1 fusion pathway model.

Collectively, we postulate that gD, gH/gL, and gB form a gD-gH/gL-gB complex that exists at a steady level, with gH/gL positioned in between gD and gB. A preassembled gD-gH/gL-gB complex in this orientation would enable efficient transmission of an activating signal from gD-receptor interaction to gH/gL to gB to trigger its fusogenic refolding (Fig. 9b). We propose that this signal transduction occurs by the following conformational cascade model. Upon receptor binding to HSV-1 gD, gD undergoes a conformational change (72) that activates the already bound gH/gL ectodomain. This causes a conformational change in the gH/gL ectodomain (38, 47) that is transmitted through the gH TMD and activates the gH CT to alter its interaction with the gB CTD (15, 63, 73). Ultimately, this triggers gB to refold and cause fusion (Fig. 9b) (15). Hence, if any of the HSV-1 gH domains are replaced, the signaling sequence from receptor to gB is disrupted and fusion cannot occur.

### Open questions.

Here, we identified steady gD-gH/gL, gH/gL-gB, and gD-gB interactions, suggesting that pairs of these interacting partners exist in complexes with one another. However, it is still undetermined whether these pairs exist independently or whether they form the gD-gH/gL-gB complex. Our fusion pathway model (Fig. 9b) favors the latter possibility, which would position the glycoproteins ideally for rapid signal transduction upon receptor binding. It is yet unclear how these complexes change during fusion. The steady interactions observed here for each interaction pair suggest that the complexes do not associate or dissociate during fusion but may, instead, change their conformation to fuse membranes. Furthermore, it is yet unclear how these glycoproteins interact on the virions. How glycoproteins are distributed on the HSV-1 surface and how they interact before and throughout the fusion process are important questions that beg in-depth investigation.

Our data support a model in which gD, gH/gL, and gB interact throughout the fusion process and that their complexes are functionally important. We also hypothesize that these complexes have important roles in membrane fusion. Nonetheless, we cannot yet rule out the possibility that the function of these complexes is something other than membrane fusion. One limitation of our study is that we do not know what fraction of the glycoproteins form complexes or whether fusion is mediated by gB within a glycoprotein complex rather than by free gB that is triggered by a transient interaction with gH/gL. However, most interaction assays, including coimmunoprecipitation, suffer from this limitation. Future single-molecule tracking experiments will be needed to resolve this question.

## MATERIALS AND METHODS

### Cells and plasmids.

CHO cells (74) were gifts from J. M. Coffin and were grown in Ham’s F-12 medium with 10% fetal bovine serum (FBS), 100 IU penicillin, and 100 μg/mL streptomycin at 37°C and 5% CO2, except when noted otherwise. Plasmids pPEP98, pPEP99, pPEP100, and pPEP101 carry the full-length HSV-1 (strain KOS) gB, gD, gH, and gL genes, respectively, in a pCAGGS vector. These plasmids were gifts from P. G. Spear (Northwestern University) (75). Plasmids RLuc8_1-7_ and Rluc8_8-11_ (encoding the *Renilla* split luciferase genes) and pBG38 (carrying the nectin-1 gene) were gifts from G. H. Cohen and R. J. Eisenberg (University of Pennsylvania) (51, 76). Plasmid pJLS11 (gB868) was generated previously (55). Plasmids for the NanoBiT interaction assay, including PRKACA-Sm and PRKAR2A-Lg positive-control plasmids, the Halo-Sm negative-control plasmid, and Sm- and Lg-BiT plasmids for tagging proteins of interest were purchased from Promega (Madison, WI) (49). All plasmids from Promega contained an HSV-TK promoter. Plasmids p85 and p25 carry the full-length EBV gH and EBV gL genes in a pCAGGS vector, respectively, and were gifts from R. M. Longnecker (Northwestern University).

### Cloning.

The cloning strategies and primers used to generate the constructs used in this paper are included in Text S1 and Table S1 in the supplemental material.

10.1128/mbio.02039-22.2 TEXT S1 Supplemental methods. Download Text S1, DOCX file, 0.02 MB. Copyright © 2022 Pataki et al. 2022 Pataki et al. https://creativecommons.org/licenses/by/4.0/ This content is distributed under the terms of the Creative Commons Attribution 4.0 International license.

10.1128/mbio.02039-22.1 TABLE S1 Primers used for cloning. Download Table S1, DOCX file, 0.03 MB. Copyright © 2022 Pataki et al. 2022 Pataki et al. https://creativecommons.org/licenses/by/4.0/ This content is distributed under the terms of the Creative Commons Attribution 4.0 International license.

### Western blotting.

Total cellular expression of NanoBiT constructs was tested using Western blotting. CHO cells were seeded at 2.5 × 10^5^ cells per well in 6-well plates. The next day, DNA constructs of interest were transfected. On day 3, cells were treated with RIPA buffer and a protease inhibitor and collected and spun down. The supernatants were mixed with SDS-PAGE loading dye and heated for 5 min at 95°C. Samples were separated by electrophoresis, transferred onto nitrocellulose membranes, and blocked. Strips of membranes were incubated with the appropriate primary antibody overnight at 4°C. On day 4, membranes were incubated with fluorescent secondary antibodies for 1 h at room temperature. Membranes were imaged using a LI-COR Odyssey imager. More detailed methods are included in Text S1 in the supplemental material.

### Flow cytometry.

Cell surface expression of gB, gH/gL, and gD constructs were evaluated using flow cytometry. CHO cells were seeded at 2.5 × 10^5^ cells per well in 6-well plates. The next day, each well was transfected with DNA constructs of interest. On day 3, cells were collected and incubated for 1 h on ice with appropriate primary antibodies. Cells were washed, incubated for 1 h on ice in the dark with secondary antibodies, and washed again. The fluorescence of the cells was determined by flow cytometry. More detailed methods are included in Text S1 in the supplemental material.

### NanoBiT interaction assay.

Interactions between glycoproteins were measured using the NanoBiT interaction assay (49). CHO cells were seeded into 6-well plates at 2.5 × 10^5^ cells per well for effector cells and 6-well plates at 2 × 10^5^ cells per well for target cells. The next day, effector cells were transfected with DNA of the two interacting partners and any remaining HSV-1 proteins required for fusion. Target cells were transfected with nectin-1 (pBG38) or pCAGGS; 4 h later, the effector cells were collected and seeded into 3 wells per condition in a 96-well plate. On day 3, the medium of the effector cells was replaced with 40 μL per well of fusion medium (Ham’s F12 with 10% FBS, penicillin/streptomycin, 50 mM HEPES) with 1:50 Endurazine luciferase substrate (Promega) added. Cells were placed in a BioTek plate reader. Luminescence measurements were taken every 2 min for 1 h at 37°C. Meanwhile, target cells were collected and resuspended in fusion medium. Then, 40 μL of target cells was added to each well of effector cells. Luminescence measurements were taken every 2 min for 7.5 or 8 h. More detailed methods are included in the Text S1 in the supplemental material.

### Cell-cell fusion assay.

Cell-cell fusion of gB, gH/gL, and gD constructs was tested using a split-luciferase assay (51). CHO cells were seeded into 3 wells per condition in a 96-well plate at 5 × 10^4^ cells per well for effector cells and 6-well plates at 2 × 10^5^ cells per well for target cells. The next day, effector cells were transfected with DNA of constructs of interest and part of a split luciferase (Rluc8_1–7_). Each well of target cells was transfected with the complementary part of the split luciferase (Rluc8_8–11_) and nectin-1. On day 3, the medium of the effector cells was replaced with 40 μL per well of fusion medium with 1:500 EnduRen luciferase substrate (Promega) added. Cells were incubated for 1 h at 37°C. In the meantime, target cells were collected and resuspended in fusion medium. Then, 40 μL of target cells was added to each well of effector cells. The plate was immediately placed in a BioTek plate reader. Luminescence measurements were taken every 2 min for 2 h followed by measurements every hour for 6 h. More detailed methods are included in Text S1 in the supplemental material.

### Statistics.

Statistical analysis was done for each experiment using GraphPad Prism 9 software. An unpaired *t test* with Welch’s correction was used to compare conditions to each other as shown.
