# Erratum for Pataki et al., “Herpes Simplex Virus 1 Entry Glycoproteins Form Complexes before and during Membrane Fusion”

**DOI:** 10.1128/mbio.03259-22

**Published:** 2022-12-06

**Authors:** Zemplen Pataki, Andrea Rebolledo Viveros, Ekaterina E. Heldwein

## ERRATUM

Volume 13, no. 5, e02039-22, 2022, https://doi.org/10.1128/mBio.02039-22. Page 17, Acknowledgments, paragraph 2, last line: “into a separate paragraph at the end of the Acknowledgment section” should be deleted.

