# Supplementary material for: Herpes Simplex Virus 1 Entry Glycoproteins Form Complexes before and during Membrane Fusion
Source: mBio. 2022 Aug 16;13(5):e02039-22. doi: 10.1128/mbio.02039-22 (PMC9600979; doi:10.1128/mbio.02039-22)
Supplement: TABLE S1 [file mbio.02039-22-s0002.docx]

**Table S1. Primers used for cloning.**

| **Primer** | **Sequence (5’-3’)** | **Primer** | **Sequence (5’-3’)** |
| --- | --- | --- | --- |
| P1 | GCGACTTTGACGAGGCCAAGCTAGCCGAGGCCAGGGAGATGATACGGTACATG | P21 | GCTCAGGGGAATTCTGGTTAATTAACGGTACCCGGGTCCCCCATGGGGAATG |
| P2 | GCCACCACCGCTCGAGAGGTCGTCCTCGTCGGCGTCAC | P22 | ACCTCCGCTCCCGCCACCACTCGAGCCTTCGCGTCTCCAAAAAAACGGGACACTTGTCCG |
| P3 | ACGAGGACGACCTCTCGAGCGGTGGTGGC | P23 | GCCTTCGTCCCTGAGGCCTCACATCGGTGCGGGGGGCAGTCT |
| P4 | TGCATGGCAGATCGCCTAGCTTAATTAACCAGAATTCCCCTGAGCTCC | P24 | ACTTGTGACTCTAAACTTGCGCGGCCAGAGAAATTCCCGTAGGATGCCAGCCAGGGCGGC |
| P5 | GACGCCGACGAGGACGACCTCTCGAGCGGTGGTGGC | P25 | ACCTCCGCTCCCGCCACCACTCGAGCCAACACTTGTGACTCTAAACTTGCGCGGCCAGAG |
| P6 | TCAAGGGCATCGGTCGACG | P26 | GCTCAGGGGAATTCTGGTTAATTAACGGTACCCGGGTCCCCCATGCAGTTG |
| P7 | AACATTTCTCTGGCCTAACTGGCCGGTACCTGAGTCTCGTTACATAACTTACGGTAAATG | P27 | GGCGGCCAGAAACCCTGCTCTTTCTTCATACAGGCCCGCAATTTCCATGACAGT |
| P8 | CAACAGTACCGGATTGCCAAGCTTCATGGTAATAGCGATGACTAATACG | P28 | AGAAAGAGCAGGGTTTCTGGCCGCCTCTGCGCT |
| P9 | ACCACCGACCTCAAGTACAACC | P29 | AAGTCCTGGCCCAGCAGACC |
| P10 | GTTGACAGGGTGCTGTATGTGGTTG | P30 | ACTCTAAACTTGCGCGGCCAGAGAAATTCCCGAACCAGAAAGATACCCAGAGCAAAAGC |
| P11 | ACATACAGCACCCTGTCAACGATATGTT | P31 | GACCTCACACGAGACAAGCTGC |
| P12 | TTGGACGATCACGTTGTCCGC | P32 | CGCTCCCGCCACCACTCGAGCCAAGGAAAAACATAACAATCTTGTGAACCAGAAAGATAC |
| P13 | CACGCAGCCCGTGGCCGCAATTGCGCCCGGGTTTCTGGCCGC | P33 | GCTCAGGGGAATTCTGGTTAATTAAGCTAGGCGATCTGCCATGGGGGGGGCTGCCGCCAG |
| P14 | GCCACCACTCGAGCCTTCGCGTCTCCAAAAAAACGG | P34 | TCCGCTCCCGCCACCACCGCTCGAGTAAAACAAGGGCTGGTGCGAGGACGGCTG |
| P15 | GCGAAGGCTCGAGTGGTGGCGGGAGCGGAGGTGGAGGGTC | P35 | CGCACCAGCCCTTGTTTTACGGCTCGAGTGGTGGCGGGAGCGGAGG |
| P16 | CATGGGGGACCCGGGTACCGTTAATTAACCAGAATTCCCCTGAGCTCCCACTTAGGCG | P36 | TATCTTATCATGTCTGCTCGAAGCGGCCGGCCGCC |
| P17 | ATCATTTTGGCAAAGAATTCGAGCTCGGTACC | P37 | GGCGTAGAGGATCGAGATCTGTACCTGAGTCTCGTTACATAACTTACGGTAAATGG |
| P18 | AACAACGTGGGGCGCAATTGCGGCCAC | P38 | GGTGGCTTTACCAACAGTACCGGATTGCCAAGCTTCATGGTAATAGCGATGACTAATACG |
| P19 | CAATTGCGCCCCACGTTGTTTTGGCAATAATCC | P39 | CTGCCATGGCGATCGCTAGCGGTGGCTTTACCAACAGTAC |
| P20 | CCAAAAAAACGGGACACTTGTCCGGAGAACCTTAACCAGAAAGATACCCAGAGC |  |  |
